# Evaluation of Phenolic Acids as Selective Bioherbicides: A Preliminary Study on Their Effects on *Ambrosia artemisiifolia* L. Germination and Soil Beneficial Bacteria

**DOI:** 10.3390/plants15071127

**Published:** 2026-04-07

**Authors:** Maja Šćepanović, Irina Tanuwidjaja, Laura Pismarović, Valentina Šoštarčić, Mirna Mrkonjić Fuka

**Affiliations:** 1 University of Zagreb Faculty of Agriculture, Division of Phytomedicine, Department of Weed Science, Svetošimunska 25, 10000 Zagreb, Croatia; lpismarovic@agr.hr (L.P.); vsostarcic@agr.hr (V.Š.); 2 University of Zagreb Faculty of Agriculture, Division of Agroecology, Department of Microbiology, Svetošimunska 25, 10000 Zagreb, Croatia; ianuwidjaja@agr.hr (I.T.); mfuka@agr.hr (M.M.F.)

**Keywords:** common ragweed, chlorogenic acid, *p*-hydroxybenzoic acid, *p*-coumaric acid, vanillic acid, phenolic acid mixture, growth promotion bacteria

## Abstract

The aim of this in vitro study was to investigate the effects of six phenolic acids applied individually and in combination at concentrations of 0–20 mM on *Ambrosia artemisiifolia* and soil bacteria. Chlorogenic acid (CGA), *p*-hydroxybenzoic acid (PHBA), and protocatechuic acid (PKA) were tested on both plants and bacteria, whereas *p*-coumaric (PCA), vanillic (VA), and ferulic (FA) acids were tested only on soil bacteria. The estimated EC_50_ for radicle inhibition were 4.9 ± 0.1 mM for PHBA, 4.1 ± 0.7 mM for CGA, 6.6 ± 0.7 mM for PKA, 10.1 ± 0.9 mM for CGA + PHBA + PKA, 4.6 ± 0.4 mM for ferulic, vanillic, and *p*-coumaric acids (FA + VA + PCA), and 2.5 ± 0.3 mM for the combination of all six phenolic acids. Bacterial strains were less susceptible to individual phenolic acids compared to their combinations. PKA and CGA showed the strongest antibacterial activity, with PKA inhibiting 78% and killing 74% of strains at ≤10 mM, while CGA inhibited 61% and killed 57%. Conversely, PCA and VA had the weakest antibacterial effects, requiring ≥20 mM for complete inhibition. Among test genera, *Stenotrophomonas*, *Bacillus*, *Peribacillus*, and *Pseudomonas* were more susceptible than *Enterobacter* and *Lelliottia*. Subinhibitory concentrations of individual phenolic acids did not affect bacterial motility, except for PKA. The study suggests that VA, PCA and FA alone or combined and PHBA alone, appear promising for weed management. Reduced herbicide strategies may safely incorporate CGA and PKA at concentrations below 2.5 mM.

## 1. Introduction

The European Commission, as part of the European Green Deal strategy, calls for a transformation of the EU economy toward a more sustainable future, with particular emphasis on reducing dependence on pesticides in agriculture. In accordance with Directive 2009/128/EC on the sustainable use of pesticides, this reduction must be achieved through the implementation of integrated pest management and other environmentally friendly practices. One promising approach is the use of secondary metabolites or allelochemicals [1], which are naturally occurring and typically degrade more rapidly than many synthetic herbicides [2]. Allelopathic crops are frequently used as cover crops or integrated into intercropping systems to suppress weed growth and reduce reliance on chemical pesticides [3].

Recent advances in compound separation and structural elucidation have enhanced the detection and characterization of allelopathic substances [4,5]. The inhibitory potential of allelopathic plants toward weed species is often correlated with phenolic compound concentration [6,7,8]. While the mechanisms of phenolic acids on weed physiology are not fully understood, existing studies show that some compounds influence seed germination, plant growth, phytohormone activity, mineral uptake, water balance, and the synthesis of specific metabolites [9]. Some phenolic acids have also been reported to inhibit the D1 protein, a key component of the photosystem II reaction center, which is essential for efficient photosynthesis [10].

Various phenolic acids, including *p*-hydroxybenzoic, protocatechuic, vanillin, salicylic, ferulic, and *p*-coumaric acids, inhibit the early growth of weeds such as *Echinochloa crus-galli*, *Galinsoga parviflora*, and *Avena fatua* [11,12]. *Ambrosia artemisiifolia* has been shown to be sensitive to *p*-coumaric, ferulic, and vanillic acids in vitro [13]. The strength of inhibition depends on concentration, chemical structure, and species [14], with higher doses generally being more effective [15,16,17]. Species-specific sensitivity has been linked to differences in seed size [18], and *A. artemisiifolia* appears more sensitive than crops like maize [13], highlighting potential weed management applications.

Bioherbicides based on phenolic acids provide an eco-friendly alternative that reduces reliance on synthetic chemicals. They help manage and mitigate resistance in weed populations, leading to long-term cost savings for farmers through decreased herbicide purchases and improved crop yields due to healthier ecosystems. It has been estimated that the financial cost of some synthetic herbicides may be higher than that of phenolic acids. Therefore, the authors suggest that reducing the herbicide dose by incorporating phenolic acids could also present an economical prospect for agricultural producers [15].

Phenolic acids may also affect non-target organisms such as soil microbiota. Their impact on microbial biomass, diversity, and activity depends on concentration, chemical structure, and plant cover [19,20,21,22,23]. Most in vitro studies focus on clinical or food-related pathogens [24,25,26,27], while effects on environmental bacterial isolates remain largely unexplored, including potential interactions of phenolic acid mixtures and strain-specific responses.

In this study, *A. artemisiifolia* was selected as a model weed due to its agricultural and public-health significance, including high allergenic pollen production and herbicide resistance [28,29,30,31]. To evaluate effects on beneficial soil microbiota, representative strains of *Pseudomonas*, *Bacillus*, *Peribacillus*, *Stenotrophomonas*, *Enterobacter*, *Lelliottia*, and *Bradyrhizobium* were included, as these genera are abundant, ubiquitous, and associated with plant growth-promoting traits. Therefore, the aim of the study is (i) to assess the inhibitory potential of chlorogenic, *p*-hydroxybenzoic, and protocatechuic acids, individually, in pairwise combinations, and with ferulic, vanillic, and *p*-coumaric acids on *A. artemisiifolia* germination, germination dynamics and early growth; (ii) to determine the minimum inhibitory and bactericidal concentrations of these phenolic acids and their mixtures against selected soil bacterial strains; and (iii) to evaluate the effects of subinhibitory concentrations on bacterial motility and morphology.

This study is the first to simultaneously investigate individual and combined phenolic acids at equivalent molecular concentrations on both *A. artemisiifolia* and soil-derived bacterial isolates. The work is timely, aligning with recent European Food Safety Agency (EFSA) recommendations on risk assessment of plant protection products on soil organisms, including microorganisms.

## 2. Results

### 2.1. Identification of the Most Sensitive Growth Parameter of Ambrosia artemisiifolia as an Indicator of Phenolic Acid Inhibitory Effects

**Figure 1:**
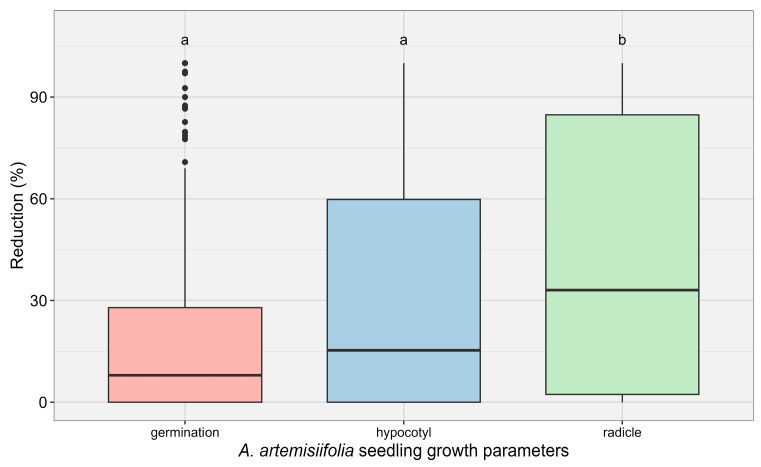
Average reduction in growth parameters in *A. artemisiifolia* treated with seven concentrations of phenolic acids applied individually or in mixtures. Vertical bars represent estimated marginal means ± standard errors. According to Tukey’s test, different lowercase letters indicate significant differences (*p* < 0.05) among the phenolic acids and applied doses. Chlorogenic acid (CGA), *p*-hydroxybenzoic acid (PHBA), and protocatechuic acid (PKA) were tested individually, as well as in mixtures: MIX PPK (combination of the three individual acids), MIX FVP (ferulic, vanillic, and *p*-coumaric acids), and MIX 6 (all six acids combined).

ANOVA revealed significant differences in the effects of phenolic acids (individual and mixtures) on germination, hypocotyl length, and radicle length (*p* < 0.001). Radicle length was the most sensitive parameter, showing on average approximately 80% inhibition and being significantly more affected than germination or hypocotyl length (Figure 1). Therefore, subsequent analyses focused on radicle inhibition.

### 2.2. Effects of Phenolic Acids on Radicle Inhibition and Germination Dynamics of Ambrosia artemisiifolia

**Figure 2:**
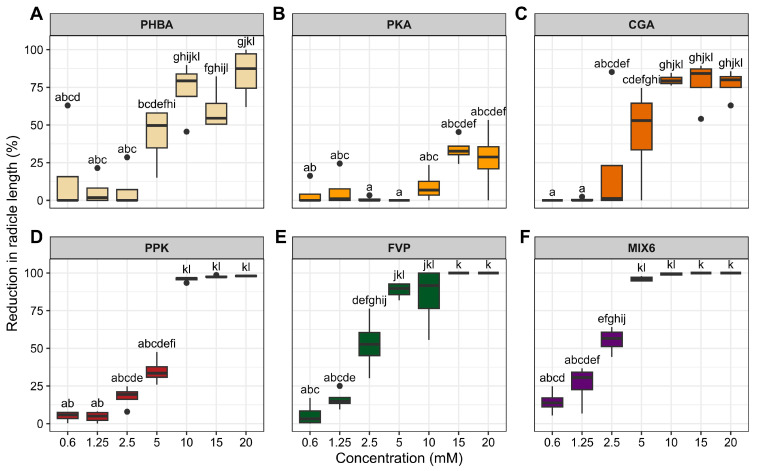
Effect of phenolic acids and their mixtures on the reduction (%) of radicle length of *Ambrosia artemisiifolia* L. The vertical bars represent the estimated marginal means + standard errors. According to Tukey’s test, different lowercase letters represent significant differences (*p* < 0.05) between the phenolic acids and the concentration applied. (**A**) *p*-hydroxybenzoic acid (PHBA), (**B**) protocatechuic acid (PKA), (**C**) Chlorogenic acid (CGA), were tested individually, as well as in mixtures: (**D**) MIX PPK (combination of the three individual acids), (**E**) MIX FVP (ferulic, vanillic, and *p*-coumaric acids), and (**F**) MIX 6 (all six acids combined).

Phenolic acids and their mixtures exerted different effects on radicle growth, with a significant interaction between acid type and concentration, indicating concentration-dependent responses (Table A2). Radicle inhibition generally increased with increasing concentration for individual acids and most mixtures (Figure 2). The MIX 6 combination (all six acids) produced the strongest inhibition (~95%) at just 5 mM and exhibited the least variability. Radicle inhibition generally increased with concentration for all individual phenolic acids and their mixtures (Figure 2).

At low concentrations (0.6–2.5 mM), radicle inhibition was generally low to moderate (0–35%), with notable variation among treatments: PHBA and CGA showed weak inhibition at these levels. In comparison, mixtures (especially MIX FVP and MIX 6) did not induce substantial inhibition below 2.5 mM. At intermediate concentrations (5–10 mM), inhibition increased markedly, with mixtures of three or six phenolic acids demonstrating significantly stronger radicle suppression (50–90%) compared to individual acids. PKA and PHBA achieved high inhibition levels at 10 mM, though variability within treatments persisted.

At the highest concentrations (15–20 mM), mixtures (MIX FVP, MIX PPK, MIX 6) consistently caused near-complete radicle inhibition (≥90%), indicating a pronounced clustering of values at the upper end of the scale. Individual phenolic acids reached complete inhibition sporadically, suggesting that mixtures may exert a synergistic or additive inhibitory effect. Tukey’s test indicated significant statistical separation among low, intermediate, and high-inhibitory concentrations (*p* < 0.05). Overall, the data demonstrate a strong concentration-dependent response, with phenolic-acid mixtures serving as the most effective inhibitors of radicle elongation.

**Figure 3:**
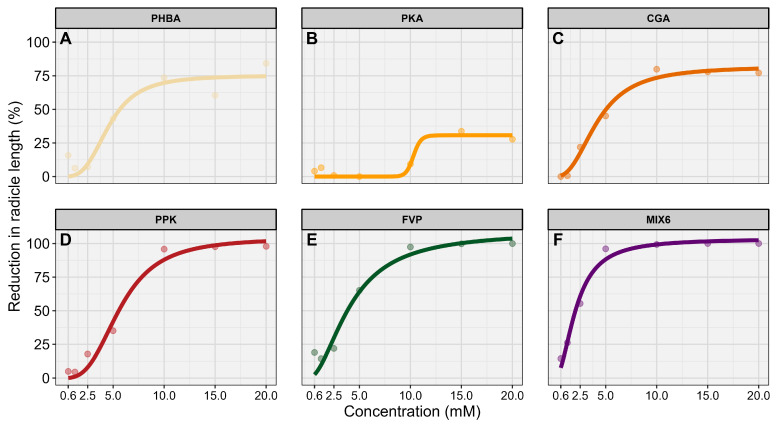
Dose–response curves showing the reduction in radicle length of *A. artemisiifolia* following exposure to individual phenolic acids and their mixtures. (**A**) *p*-hydroxybenzoic acid (PHBA), (**B**) protocatechuic acid (PKA), (**C**) Chlorogenic acid (CGA), were tested individually, as well as in mixtures: (**D**) MIX PPK (combination of the three individual acids), (**E**) MIX FVP (ferulic, vanillic, and *p*-coumaric acids), and (**F**) MIX 6 (all six acids combined). Points represent observed values, and solid lines indicate fitted dose–response curves.

Dose–response curves were generated for CGA, PHBA, and PKA applied individually, for their combined application (MIX PPK), and for mixtures including these acids with ferulic, vanillic, and *p*-coumaric acids (MIX 6) and for the mixture of ferulic, vanillic, and *p*-coumaric acids alone (MIX FVP). The estimated EC_50_ values were 4.9 ± 0.1 mM for PHBA, 4.1 ± 0.6 mM for CGA, 10.1 ± 0.9 mM for PKA, 5.8 ± 0.6 mM for MIX PPK, 4.6 ± 0.7 mM for MIX FVP, and 2.5 ± 0.3 mM for MIX 6 (Figure 3).

**Figure 4:**
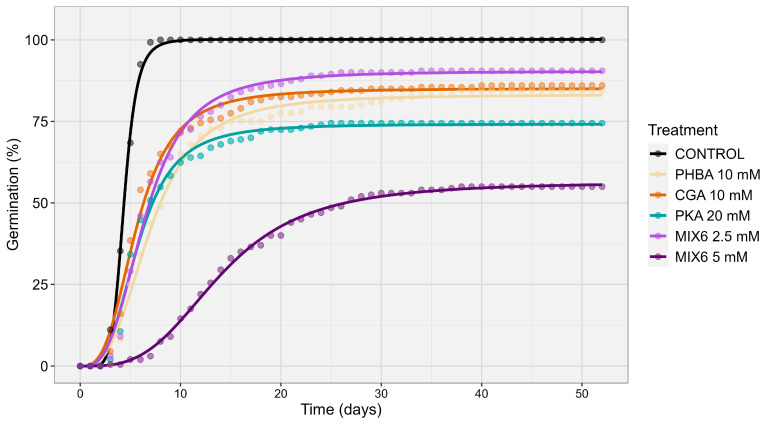
Dose–response curves showing the germination dynamic of *A. artemisiifolia* following exposure to individual phenolic acids and their mixtures. Curves were fitted using a non-parametric time-to-event model. The x-axis represents time (days), and the y-axis represents cumulative germination (%) of *A. artemisiifolia* seeds. Chlorogenic acid (CGA), *p*-hydroxybenzoic acid (PHBA), and protocatechuic acid (PKA) were tested individually, as well as in mixtures, MIX 6 (*p*-hydroxybenzoic acid (PHBA), protocatechuic acid (PKA), chlorogenic acid (CGA), ferulic acid (FA), *p*-coumaric acid (PCA), vanillic acid (VA).

Analysis using the non-parametric time-to-event model and permutation tests revealed significant differences in the germination dynamics of *A. artemisiifolia* seeds treated with CGA, PHBA and PKA, along with their mixtures (MIX 6). These phenolic acids and their combinations differentially influenced germination rates, as indicated by T_50_, T_90_ values, maximum germination (Gmax), curve slope, and area under the curve (AUC) (Figure 4). All phenolic acid treatments produced similar shallow germination curves, while the control exhibited a significantly steeper slope, indicating faster and more synchronized germination (Table A3). This demonstrates that phenolic acids consistently slowed and flattened germination dynamics compared to untreated seeds. All treatments significantly reduced total germination rates compared to controls, with notable differences in inhibition levels, especially between single acids and mixtures (Table A4). Among individual compounds, PKA (10 mM) had the strongest inhibitory effect, reducing GRmax to 74% of the control. The greatest inhibition occurred with all acids combined, lowering GRmax to 56% of the control and resulting in a reduced AUC, indicating weaker overall germination dynamics than both the control and single-acid treatments (Table A5).

All phenolic acids and their mixtures significantly slowed the germination dynamics of *A. artemisiifolia* seeds compared with untreated seeds (Figure 4). Estimated T_50_ values showed that untreated seeds required only 4.3 days to reach 50% germination, whereas seeds treated with phenolic acids required between 5.6 and 14.0 days, with significant differences among the phenolic acid treatments. Among the individually applied compounds, *p*-hydroxybenzoic acid (PHBA, 10 mM) produced the strongest delay (T_50_ = 7 days), significantly higher than chlorogenic and protocatechuic acids. The greatest inhibition of germination speed occurred again when the mixture of all six phenolic acids was applied at 5 mM, requiring 14.0 days to reach 50% germination.

### 2.3. Genotypic Characterisation and Strain Diversity of Soil-Derived Bacteria

**Figure 5:**
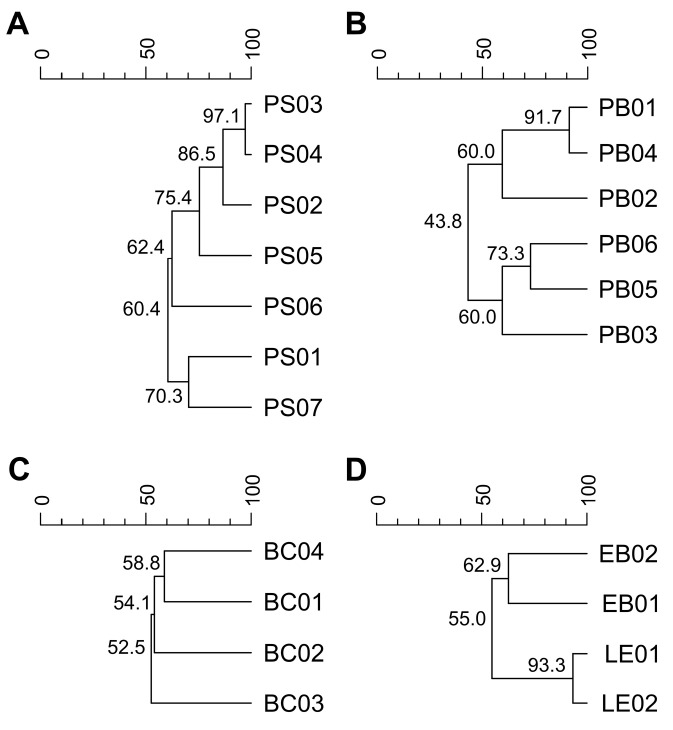
Strain diversity of selected soil-derived bacteria based on (GTG)5 rep-PCR profiles. The analysis includes isolates belonging to the genera (**A**) *Pseudomonas*, (**B**) *Peribacillus*, (**C**) *Bacillus*, and (**D**) *Enterobacter* and *Lelliottia*.

Rep-PCR profiles confirmed that all soil-derived bacteria belonging to the genera represented with more than one isolate (*Pseudomonas*, *Bacillus*, *Peribacillus*, *Enterobacter*, and *Lelliotia*) were distinct strains (Figure 5).

The diversity varied among the strains, with *Peribacillus* being the most diverse, with between-strain similarity ranging from 43.8 to 91.7%, followed by *Pseudomonas* strains, whose similarity ranged from 60.4 to 97.1%. In contrast, *Bacillus* strains were relatively similar (52.5–58.8%), while *Enterobacter* and *Lelliottia* strains showed 62.9 and 93.3% similarity, respectively.

### 2.4. The Susceptibility of Selected Soil-Derived Strains to Phenolic Acids

The MIC values corresponded to the MBC values, except for PHBA, PKA, CGA and VA against strain PS07, where the MBC values were twice as high as the MIC values (Table 1). Overall, the susceptibility to phenolic acids, whether applied individually or in combinations, varied among strains regardless of the genus they belonged to. However, a similar trend was observed across all strains, with lower susceptibility to individual phenolic acids compared to their combinations. Among the individual phenolic acids, protocatechuic (PKA) and chlorogenic acid (CGA) showed the strongest inhibitory activity, which was reflected by the lowest MIC values observed. The MIC and MBC values for PKA ranged from 2.5 to 40 mM, with 78% of strains inhibited, and 74% killed at concentrations ≤ 10 mM. CGA exhibited inhibitory and bactericidal activity within the same range as PKA; however, its antibacterial activity was less pronounced, inhibiting 61% and killing 57% of strains at concentrations ≤ 10 mM. PHBA showed moderate antibacterial activity, inhibiting and killing 43% and 39% of strains, respectively. In contrast, PCA and VA showed the lowest overall antibacterial activity, with complete inhibition or bacterial death observed only at concentrations ≥ 20 mM. Finally, MIX6 was identified as the most potent antibacterial agent, exhibiting inhibitory and bactericidal activity at concentrations ≤ 5 mM. This combination inhibited or killed 74% of strains at 2.5 mM and 26% of strains at 5 mM. MIX6 was followed by the PPK combination, with MIC/MBC values ranging from 2.5 to 10 mM, where 48% of strains were inhibited or killed at 2.5 and 5 mM, and only 4% of strains at 10 mM. Consistent with the antibacterial activity of its individual components, FVP exhibited the weakest antibacterial activity among the tested combinations, with MIC/MBC values ranging from 2.5 to 20 mM. The majority of strains were inhibited or killed at higher FVP concentrations (10 mM: 65%; 20 mM: 26%), whereas only 4% of strains were affected at low concentrations (2.5 mM: 4%; 5 mM: 4%) (Table 1).

**Figure 6:**
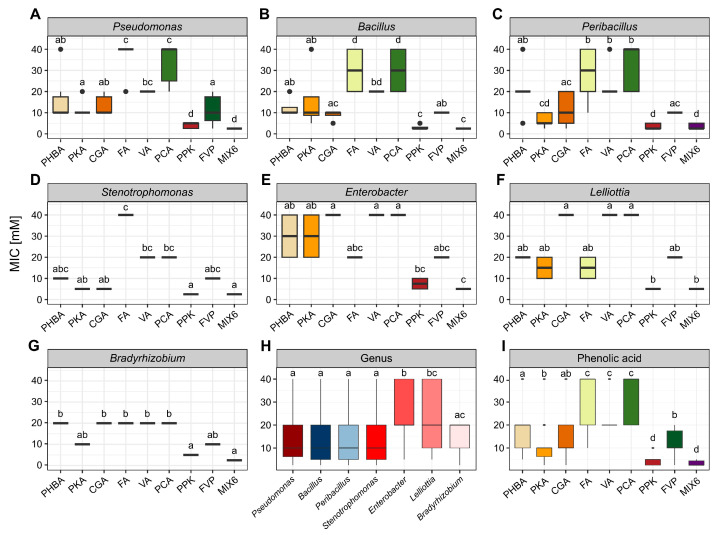
Antibacterial activity based on minimum inhibitory concentrations (MIC) of phenolic acids, applied individually or in combinations against soil-derived bacteria. The analysis includes bacterial strains belonging to the genera (**A**) *Pseudomonas* sp., (**B**) *Bacillus* sp., (**C**) *Peribacillus* sp., (**D**) *Stenotrophomonas* sp., (**E**) *Enterobacter* sp., (**F**) *Lelliottia* sp., and (**G**) *Bradyrhizobioum* sp. Panels (**H**) and (**I**) depict genus-dependent and phenolic acid-dependent susceptibility of selected soil-derived bacteria, respectively. According to Tukey’s test, different lowercase letters represent significant differences (*p* < 0.05) between the phenolic acids and the concentration applied.

Susceptibility to phenolic acids at the genus level was consistent with strain-dependent patterns, with all genera being less susceptible to individual phenolic acids than to their combinations. Although the antibacterial activity of individual phenolic acids varied more on the genus level than at the strain level, the overall trend was the same. PKA and CGA (MIC: 2.5–40 mM) were the most effective inhibitors, whereas PCA and VA (20–40 mM) were the least effective. Moreover, the combinations PPK and MIX 6 exhibited again, higher inhibitory potential than the FVP combination (Figure 6A–G).

The mean MIC values averaged across genera (Figure 6H) showed that *Stenotrophomonas* (12.8 ± 11.8 mM), *Bacillus* (14.8 ± 11.9 mM), *Peribacillus* (15.6 ± 12.8 mM), and *Pseudomonas* (16.5 ± 12.9 mM) exhibited similar susceptibility and were significantly more susceptible to phenolic acids than *Enterobacter* (25.8 ± 13.9 mM) and *Lelliottia* (22.2 ± 14.0 mM) (Kruskal–Wallis: *p* < 0.001; Dunn’s test with Benjamini–Hochberg correction: all pairwise comparisons *p* < 0.05). The mean MIC values averaged over phenolic acids (Figure 6I) confirmed MIX6 (3.2 ± 1.1 mM) and PPK (4.0 ± 1.8 mM) as the most effective inhibitors among all tested phenolic acids, and PCA (33.3 ± 9.6 mM) and FA (29.6 ± 11.3 mM), together with VA (24.3 ± 8.3 mM) as the least effective (Kruskal–Wallis: *p* < 0.001; Dunn’s test with Benjamini–Hochberg correction: all pairwise comparisons *p* < 0.05).

**Figure A1:**
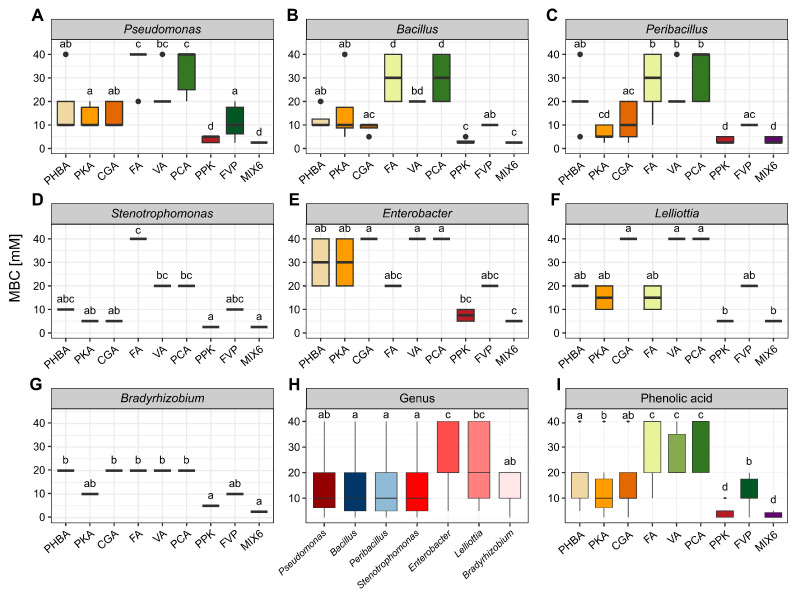
Antibacterial activity based on minimum bactericidal concentrations (MBC) of phenolic acids, applied individually or in combinations against soil-derived bacteria. The analysis includes bacterial strains belonging to the genera (**A**) *Pseudomonas* sp., (**B**) *Bacillus* sp., (**C**) *Peribacillus* sp., (**D**) *Stenotrophomonas* sp., (**E**) *Enterobacter* sp., (**F**) *Lelliottia* sp., and (**G**) *Bradyrhizobium* sp. Panels (**H**,**I**) depict genus-dependent and phenolic acid dependent susceptibility of selected soil-derived bacteria, respectively. Since all MBC values, except for the *Pseudomonas* strain PS07, correspond to the MIC values, MBC based antibacterial activity does not differ from MIC (Figure 6), except for (**A**) *Pseudomonas* sp., (**H**) genus-dependent, and (**I**) phenolic acid dependent susceptibility of selected soil-derived bacteria to *p*-hydroxybenzoic acid (PHBA), protocatechuic acid (PKA), chlorogenic acid (CGA), and vanillic acid (VA). According to Tukey’s test, different lowercase letters represent significant differences (*p* < 0.05) between the phenolic acids and the concentration applied.

Finally, since all MBC values, except for one, correspond to the MIC values, the previously described trends and patterns in strain- and genus-dependent susceptibility, as well as antibacterial activity based on MIC values, were the same for MBC values (Figure A1).

### 2.5. Effect of Sub-Inhibitory Concentrations of Phenolic Acids on the Motility of Soil-Derived Strains

An overview of the effects of phenolic acids, applied individually or in combination, on the motility of selected soil-derived strains is summarized in Table 2. The effect of sub-inhibitory concentrations of phenolic acids and their combinations on non-motile strains was not assessed.

Natural motility varied among the genera. *Bradyrhizobium* sp. exhibited slow, barely detectable movement, whereas the motility in other genera varied from moderate in Enterobacterales and *Peribacillus* sp. to very fast for *Pseudomonas* sp. Notably, several strains belonging to genera typically considered motile, such as *Pseudomonas* and *Stenotrophomonas*, were non-motile prior to the treatment with phenolic acids. However, loss of motility during cultivation under laboratory conditions has been reported [32,33].

Overall, individual phenolic acids at sub-inhibitory concentrations did not affect the motility of soil-derived strains, except PKA, which inhibited motility in strains PB01 and BR01. In contrast, the phenolic acid combinations PPK and MIX6 exhibited a deleterious effect on the motility, rendering all treated strains non-motile. FVP exhibited a mixed effect on the bacterial motility, inhibiting the motility in 36% of naturally motile strains, mainly from the *Pseudomonas*, *Stenotrophomonas*, and *Bradyrhizobium* genera, while having no effect on motility in Enterobacterales strains.

## 3. Discussion

Interest in phenolic acids has grown due to their chemical diversity, biological activity, and advances in separation techniques [4,5]. Stricter regulations on synthetic herbicides have heightened the search for eco-friendly weed control alternatives. While phenolic acids are a primary focus, other viable alternatives like terpenes, essential oils, and plant extracts also contribute to weed control. For example, carvacrol, a monoterpene from *Satureja montana* essential oils, has shown significant phytotoxic effects on various weed species during germination and early growth [34]. Additionally, several allelochemicals in *Parthenium hysterophorus* L., including eight amino acids and seven phenolic compounds, have demonstrated inhibitory effects on *Cyperus iria* L., comparable to synthetic herbicides like glyphosate and glufosinate-ammonium [35]. Furthermore, water extracts from allelopathic cover crops have shown significant inhibitory effects on weed growth in various in vitro studies [36,37]. This study emphasizes phenolic acids, highlighting their established allelopathic activity against *A. artemisiifolia* [13,15], as well as their physicochemical properties, availability, safety, and potential for synergistic combinations with other compounds.

This in vitro study aimed to assess the herbicidal potential of chlorogenic, *p*-hydroxybenzoic, and protocatechuic acids, applied individually or in combination, against *Ambrosia artemisiifolia*. Additionally, it evaluated the effects of these compounds on soil microorganisms, particularly plant growth-promoting bacteria (PGPB).

### 3.1. Effect of Phenolic Acids on Ambrosia artemisiifolia

The results demonstrate that the inhibitory activity of chlorogenic, *p*-hydroxybenzoic, and protocatechuic acids is strongly concentration-dependent. At concentrations ≤ 5 mM, all three phenolic acids caused only low levels of radicle growth inhibition (0–45%). A significant increase in inhibitory activity was observed for chlorogenic and *p*-hydroxybenzoic acids at concentrations ≥10 mM (60–84%). These patterns align with previous in vitro studies reporting a similarly pronounced concentration-dependent inhibition of *A. artemisiifolia* [31] and other weed species [10,16,17].

The results further show that the magnitude of inhibition depends on the specific phenolic acid. Protocatechuic acid caused only weak inhibition across all tested concentrations, with a maximum of 33.7%, unlike chlorogenic or *p*-hydroxybenzoic acid. A log–logistic dose–response model confirmed these differences, yielding lower EC_50_ values for chlorogenic acids and *p*-hydroxybenzoic (4.1 ± 0.6 mM and 4.9 ± 0.1 mM, respectively) compared with the higher EC_50_ for protocatechuic acid (10.1 ± 0.9 mM) (Figure 3). Similar conclusions were reported in previous work on *A. artemisiifolia*, where *p*-coumaric acid showed stronger inhibitory activity than vanillic or ferulic acid [13].

These findings emphasize the importance of evaluating phenolic acids individually to identify the most effective candidates for biological weed control. In this study, chlorogenic acid produced the strongest inhibition of radicle growth (Figure 2). Chlorogenic acid has previously been isolated as a potent natural herbicide from *Artemisia argyi*, and microstructural analyses revealed epidermal cell shrinkage, reduced chloroplast abundance, and chloroplast degradation as key drivers of leaf withering and biomass loss in *Setaria viridis* [38]. Similarly, *p*-hydroxybenzoic acid has been shown to strongly inhibit PSII photochemistry in *Rumex acetosa*, with the greatest effects reported at 1.5 mM [39]. In the present study, *p*-hydroxybenzoic acid at 10 mM produced the strongest delay in *A. artemisiifolia* germination (Figure 4). Because delayed germination can greatly reduce seedling establishment under field conditions [40], this compound may be particularly promising for weed management.

Given that additive or synergistic interactions among allelopathic compounds can yield stronger inhibition than individual compounds, even at lower doses [41], several phenolic mixtures were tested. The results clearly show that mixtures containing three or six phenolic acids were substantially more inhibitory than individual compounds. This effect was most pronounced for the mixture containing all six phenolic acids, which caused nearly complete inhibition of radicle growth (>95%) at 5 mM and had an EC_50_ of 2.5 ± 0.3 mM. This mixture (5 mM) also produced the greatest delay in germination, requiring 14.0 days to reach 50% germination. These findings align with numerous studies showing that mixtures of phenolic acids or aqueous extracts containing multiple allelopathic compounds exhibit stronger phytotoxicity than individual constituents [11,15,42]. Such synergistic effects likely contribute to the growth-inhibition patterns observed in both laboratory and field settings [43]. In addition, under field conditions, phenolic allelochemicals typically occur as mixtures rather than as isolated compounds, and such mixtures generally exhibit stronger allelopathic activity than their individual components [44]. This enhanced activity may be attributed to the ability of different compound groups to act on multiple physiological targets [45].

However, despite their higher phytotoxicity, the complex interactions among diverse bioactive compounds in phenolic mixtures make them less suitable candidates for bioherbicide development. Moreover, identifying an effective individual phenolic compound is essential when developing strategies that combine reduced doses of conventional herbicides with phenolic acids [13].

For the noxious weed *A. artemisiifolia*, the present study identified *p*-hydroxybenzoic and chlorogenic acids as promising candidates, whereas previous research highlighted *p*-coumaric acid [13]. To progress toward field application, it is crucial to assess and minimize any potential adverse effects of these compounds on beneficial soil microbiota. To the best of our knowledge, no comprehensive in vitro study that integrates the evaluation of the effect of phenolic acids on germination dynamics, and early growth of weeds such as *A. artemisiifolia* weeds, and environmental isolates has been conducted.

### 3.2. Effect of Phenolic Acids on Soil-Derived Bacterial Strains

The inclusion of multiple soil-derived strains from different genera (*Pseudomonas*, *Bacillus, Peribacillus*, *Enterobacter*, and *Lelliottia*) in this study, as confirmed by rep-PCR genotyping, ensured a more realistic representation of natural variability in soil environments [46,47]. Consequently, the observed differences in susceptibility to phenolic acids among the selected strains likely reflect inherent strain-level variability rather than methodological bias. The strain-specific response to the phenolic acids is consistent with well-known heterogeneity among strains within the same species and genus, as bacterial stress responses, such as membrane adaptation, efflux capacity, metabolic detoxification, and repair pathways, often vary at the strain level [48,49]. Furthermore, this study has shown that the antibacterial activity of individual phenolic acids varied markedly, with protocatheutic (PKA) and chlorogenic acid (CGA) showing the strongest effects, inhibiting or killing most strains at ≤10 mM. In contrast, *p*-coumaric acid (PCA) and vanillic acid (VA) were the least effective, causing complete inhibition or bacterial death only at concentrations ≥ 20 mM. Observed differences in antibacterial activity could stem from variations in the chemical structure of phenolic acids. For example, the number and position of hydroxyl and methoxyl groups on the aromatic ring influence membrane disruption, enzymatic interference, and oxidative stress induction, which consequently affect the antimicrobial efficacy of phenolic acids against bacterial strains, thus explaining the stronger activity of compounds like *p*-coumaric and chlorogenic acids compared to simpler phenolics [50].

In contrast to individual application, combinations of phenolic acids were consistently more potent regardless of strain or genus. The PPK and MIX6 were the most effective combinations, with PPK inhibiting or killing 48% of strains at 2.5–5 mM, and MIX6 74% at 2.5 mM and 26% at 5 mM. This enhanced activity likely stems from synergistic interactions among the individual components, where multiple compounds act through complementary mechanisms, such as simultaneous disruption of cell membranes, inhibition of key enzymes, and induction of oxidative stress, thus resulting in greater antibacterial efficacy than predicted from their individual antibacterial activities [51].

The results of this study demonstrated that the inhibitory and bactericidal effects of phenolic acids are concentration-dependent, which is consistent with previous studies. For example, low concentrations of 2,4-di-tert-butylphenol, vanillic acid [21], and *p*-coumaric acid [19] stimulate microbial biomass development and microbial activity, whereas higher concentrations inhibit bacterial growth. This can be attributed to the mechanism of action of phenolic acids, which primarily damages bacterial cell membranes. Namely, at higher concentrations, phenolic acids cause more severe membrane disruption, alter membrane permeability, and ultimately lead to cell lysis [52]. Other studies have shown that the concentration ranges required for inhibition can vary substantially. For example, the minimum inhibitory concentrations of vanillic acid against *P. aeruginosa* ranged from 583.3 to 1333.3 µg/mL, corresponding to 3.2–7.4 mmol/L [27]. Surprisingly, these inhibitory concentrations are consistently lower than the MIC and MBC values determined for the strains used in this study, which were isolated from natural ecosystems. Bacterial isolates from natural environments are generally considered more sensitive to antimicrobial compounds than clinical isolates, which, due to continuous exposure to various antimicrobial agents, tend to exhibit higher resistance [53,54]. However, species of the genera *Pseudomonas* and *Bacillus* isolated from natural ecosystems are also capable of degrading phenolic acids [55,56,57], which may explain why higher concentrations of particular phenolic acids are required for their inhibition.

In addition, this study demonstrated genus-dependent resilience to phenolic acids, with *Enterobacter* and *Lelliottia* being the most resilient. These genera, belonging to the order Enterobacterales, known for their robust Gram-negative outer membrane and adaptive transport systems, which are responsible for greater intrinsic tolerance to phenolic compounds. Specifically, membrane porins and multidrug efflux pumps (e.g., AcrAB–TolC and related systems) can reduce intracellular accumulation of phenolics, thus mitigating their toxic effects and the associated oxidative stress from hydroxyl radical generation. For example, Sui et al. [58] demonstrated that the enhanced expression of porins and efflux systems in *Escherichia coli* and related taxa increases the tolerance to various phenolic compounds by inhibiting intracellular toxicity pathways. In contrast, many soil isolates of *Pseudomonas* and Gram-positive genera such as *Bacillus* and *Peribacillus* are susceptible to phenolics that disrupt cellular membranes and metabolic processes, unless they possess specialized degradation pathways, such as specific dioxygenases or hydroxylases capable of breaking down allelochemicals. While some *Pseudomonas* strains can degrade phenolic acids, these mechanisms are often strain-specific and not expressed across the whole genus [55].

Individual phenolic acids did not affect the motility at sub-inhibitory concentrations, except for PKA, which inhibited the motility in strains PB01 and BR01. In contrast, combinations such as PPK and MIX6 resulted in complete loss of motility in all examined strains, whereas FVP affected only 36% of motile strains, primarily *Pseudomonas*, *Stenotrophomonas*, and *Bradyrhizobium*. These observations suggest that phenolic acid combinations at low concentrations disrupt motility through multiple mechanisms, potentially interfering with flagellar biosynthesis or other motility-associated systems, disrupting intercellular communication (quorum sensing), and inducing bacterial cell elongation, without causing complete growth inhibition [59].

In this study, phenolic acids were tested at concentrations up to 20 mM, significantly higher than the typical levels found in natural or agricultural soils, which range from 0.01 to 0.5 μmol g^−1^ soil [60]. These concentrations are much lower than those required to inhibit germination and seedling growth. The bioavailability of phenolic acids in soil is strongly influenced by physicochemical factors such as soil type, pH, mineral composition, and interactions with other compounds. For instance, phenolic acids like *p*-coumaric and ferulic acid are rapidly sorbed onto soil particles, often >80% within hours [61], forming complexes with clay minerals and metal ions that limit their mobility and uptake by plants. Due to these processes, it is unlikely that phenolic acids can be transported far from their origin or maintained at phytotoxic levels in soil solution for extended periods. Given this complexity and the limited bioavailability of phenolic acids in soil, future research will focus on evaluating their effects via foliar application, allowing for more controlled and physiologically relevant exposure. However, regardless of the application method, validation under realistic environmental conditions remains essential.

While the in vitro results provide valuable insights into their potential efficacy against *Ambrosia artemisiifolia* and soil bacteria, field studies are essential to assess the practical applicability, effectiveness, and environmental impact of these compounds in diverse agricultural settings. Conducting such studies will help to understand the interactions between these phenolic acids, the target weed, and the surrounding ecosystem, ultimately leading to more sustainable weed management strategies.

## 4. Materials and Methods

### 4.1. Evaluation of the Inhibitory Effects of Phenolic Acids on the Growth Parameters of Ambrosia artemisiifolia

#### 4.1.1. Plant Material

Mature seeds of *Ambrosia artemisiifolia* were collected in autumn 2024 in Croatia, near Nova Gradiška (45°51′05.2″ N, 16°10′34.1″ E). Seeds were cleaned and stored in paper bags. Due to the primary dormancy of *A. artemisiifolia* seeds, the seeds were cold stored at 4 °C [15] and germination tests were performed periodically until germination reached at least 70%, which was the threshold required to start the experiment. Before the experiments were initiated, seeds were manually calibrated, i.e., sorted according to size and color, and empty seeds were removed by gently pressing them with tweezers and discarding those that felt soft [62]. Only uniform seeds without any visible signs of predator damage were used in the experiment.

#### 4.1.2. Phenolic Acids Solutions

Reference standards of the six phenolic acids were obtained from Sigma-Aldrich (Germany Steinheim,): ferulic acid (FA; trans-4-hydroxy-3-methoxycinnamic acid), vanillic acid (VA; 4-hydroxy-3-methoxybenzoic acid), *p*-coumaric acid (PCA; trans-4-hydroxycinnamic acid), protocatechuic acid (PKA; 3,4-dihydroxybenzoic acid), *p*-hydroxybenzoic acid (PHBA), and chlorogenic acid (CGA).

The phenolic acids were dissolved individually or in mixtures in distilled water, sonicated (35 kHz, 80 °C; Sonorex TK 52, Bandelin, Berlin, Germany), and prepared as 20 mM stock solutions. Working solutions were then adjusted to seven final concentrations: 0, 1.25, 2.5, 5, 10, 15, and 20 mM.

The phenolic acid mixtures included: MIX FVP (FA + VA + PCA), MIX PPK (PHBA + CGA + PKA), and MIX 6 (FA + VA + PCA + PHBA + CGA + PKA). All solutions were supplemented with 0.5% DMSO to improve solubility. Preliminary tests confirmed that 0.5% DMSO had no inhibitory effect on *A. artemisiifolia* germination. All six phenolic acids and their mixtures were used to assess effects on the soil microbiota, whereas chlorogenic, protocatechuic, and *p*-hydroxybenzoic acids, individually or in mixtures, were used in the in vitro experiment with *A. artemisiifolia*, since the effects of vanillic, *p*-coumaric, and ferulic acids on this weed species had already been evaluated [13].

#### 4.1.3. In Vitro Assay on *A. artemisiifolia*

For each treatment (phenolic acids and control), 50 seeds of *A. artemisiifolia* were placed on filter paper in sterilized 90 mm Petri dishes, and 4 mL of the test solution was added. Distilled water served as the control. Each treatment (PKA, CGA, PHBA, MIX FVP, MIX PPK, MIX 6, control) at each concentration (0–20 mM) was replicated four times, and the entire experiment was repeated twice. Petri dishes were sealed with Parafilm and incubated in a climate chamber (HPP 108, Memmert, Schwabach, Germany) under conditions optimal for the early growth of *A. artemisiifolia*: 12/12 h photoperiod, 25 °C/15 °C day/night temperature, 70% relative humidity, and 40–50 µmol m^−2^ s^−1^ LED light.

After 10 days, the germination percentage, hypocotyl length, and radicle length of 10 seedlings per dish were measured. Seeds were considered germinated when the radicle exceeded 1 mm in length. Inhibition (%) of germination and seedling growth was calculated using Abbott’s formula [63].

To determine germination dynamics (speed), seeds of *A. artemisiifolia* treated with phenolic acids and untreated seeds (control) were monitored daily. Germination was recorded until no new germinated seeds were observed for 10 consecutive days, resulting in a total observation period of 53 days. Concentrations of phenolic acids and mixtures used for the germination-dynamics experiment were selected based on the results of radicle-length inhibition and the ED_90_ value determined for each treatment: 10 mM (PHBA, CGA), 20 mM (PKA), and 2.5 and 5 mM for the mixture of all six phenolic acids.

### 4.2. Assessment of the Inhibitory Effects of Phenolic Acids on Soil-Derived Bacterial Isolates

#### 4.2.1. Origin and Identification of Bacterial Isolates

The isolates belonging to the genera *Pseudomonas* (*n* = 7), *Bacillus* (*n* = 4), *Peribacillus* (*n* = 6), *Stenotrophomonas* (*n* = 1), *Enterobacter* (*n* = 2), *Lelliottia* (*n* =2) and *Bradyrhizobium* (*n* = 1) were selected as model organisms that represent the most important soil-derived beneficial bacteria known to harbor plant growth promoting traits. All bacterial isolates used in this study are an integral part of the microorganism collection of the University of Zagreb Faculty of Agriculture Department of Microbiology. They were previously identified at the genus or species level using matrix-assisted laser desorption/ionisation time-of-flight mass spectrometry (MALDI-TOF MS, Brucker Daltonics, Bremen, Germany). Mass spectra were processed using the Biotyper software (version 4.1, Bruker Germany Daltonics), and isolate identification was based on the similarity of the mass spectrum of the isolate to a reference spectrum.

#### 4.2.2. DNA Extraction and Genotyping of Bacterial Isolates

To assess the diversity of strains used in this study, the bacterial isolates of the genera *Pseudomonas*, *Bacillus*, *Peribacillus*, *Stenotrophomonas*, *Enterobacter*, *Lelliottia* and *Bradyrhizobium* (*n* = 23) were genotyped. First, template DNA was extracted from all isolates using a 10.0% (*w*/*v*) Chelex^®^ 100 Resin solution (Bio-Rad Laboratories Inc., Hercules, CA, USA). In brief, bacterial biomass was prepared by streaking isolates on tryptic soy agar (TSA; Biolife, Milano, Italy), followed by aseptic inoculation of a single colony into 1.5 mL of tryptic soy broth (TSB; Biolife, Milano, Italy). After overnight incubation at 30 °C, bacterial cells were harvested by centrifugation (5417R, Eppendorf AG, Leipzig, Germany) at 16,000× *g* for 3 min, and pellets were resuspended in 200 µL of 10.0% (*w*/*v*) Chelex^®^ 100 Resin solution. The resulting suspensions were thoroughly homogenised by vortexing and incubated in a thermomixer (ThermoMixer C, Eppendorf AG, Leipzig, Germany) at 56 °C for 20 min with continuous mixing at 300 rpm. The samples were then vortexed and incubated for an additional 80 min at 100 °C with continuous mixing at 100 rpm. Finally, the Chelex^®^ 100 Resin was separated from the DNA-containing supernatant by centrifugation at 14,000× *g* for 2 min. Genomic DNA was quantified using the Qubit™ 2.0 Fluorometer (Life Technologies, Carlsbad, CA, USA) and the Qubit™ dsDNA HS Assay Kit (Life Technologies, Carlsbad, CA, USA). All DNA samples were diluted to a final concentration of 20 ng/µL in molecular biology grade water (Sigma-Aldrich, Steinheim, Germany).

Genotyping was performed by repetitive element PCR (rep-PCR) using the (GTG)_5_ primer [64]. PCR amplification, electrophoretic separation, and visualization of the PCR products were carried out according to the protocol described by. Domig et al. [65].

Rep-PCR fingerprints were analysed using BioNumerics software version 7.6.1. (Applied Maths, Sint-Martens-Latem, Belgium). The genetic similarity among the isolates was calculated based on the Dice coefficient. Isolates were then clustered using the unweighted paired group arithmetic average (UPGMA) method. Dendrograms were generated using a 1.0% tolerance level and a 0.5% optimization. Separate dendrograms were constructed only for genera with more than one isolate (*Pseudomonas*, *Bacillus*, *Peribacillus*, and members of the Enterobacterales order (*Enterobacter* and *Lelliottia*), since strain differentiation was unnecessary for genera represented by a single isolate (*Stenotrophomonas* and *Bradyrhizobium*). Based on the resulting profiles, the isolates showing less than 100% similarity (cut-off threshold for strain differentiation) were considered distinct strains.

#### 4.2.3. Determination of Minimal Inhibitory and Minimal Bactericidal Concentrations

The susceptibility of selected soil-derived strains to individuals and combinations of phenolic acids was quantified by assessing the minimum inhibitory concentration (MIC) and minimum bactericidal concentration (MBC). MIC was determined using the broth microdilution method, where each phenolic acid and its combinations were serially diluted (1:2) in a sterile liquid medium. Muller-Hinton broth (MHB; Biolife, Italy, Milano,) was used for all genera except for *Bradyrhizobium*, which were grown in yeast mannitol broth (YMB, HiMedia, Mumbai, India).

First, the stock solutions (80 mM) were prepared by dissolving reference standards (Sigma-Aldrich, Germany, Steinheim) of individual phenolic acid PHBA, PKA, CGA, FA, VA, PCA and their combinations (PHBA + PKA + CGA (PPK), FA + VA + PCA (FVP), and mix of all six phenolic acids (MIX6) in sterile MHB or YMB, depending on the strain tested. In the phenolic acid combinations, the concentration of each component was 80 mM. To facilitate the solubility of poorly soluble phenolic acids in water, up to 2% (*v*/*v*) of dimethyl sulfoxide (DMSO; Sigma-Aldrich, Steinheim, Germany) was added to the stock solutions. This resulted in a final DMSO concentration of up to 1% in the microtiter plates, a level that does not affect bacterial growth [66], as confirmed in our study.

MIC assays were performed in 96-well microtiter plates, where the stock solutions of phenolic acids and their combinations were diluted to final concentrations ranging from 0.6 to 40 mM. The bacterial inocula were prepared by suspending single colonies in sterile saline solution (0.85% NaCl; VWR Chemicals, Leuven, Belgium) until a turbidity corresponding to a 0.5 McFarland standard (approximately 1.5 × 10^8^ CFU/mL) was reached. Each inoculum was further diluted to 1.5 × 10^6^ CFU/mL, and 10 µL of the diluted bacterial suspension was added to each well, resulting in a final concentration of 1.0 ×10^5^ CFU/mL per well. Each phenolic acid-strain combination was tested in two independent replicates. Controls included inoculated MHB or YMB without phenolic acids, and the same media supplemented with DMSO at a final concentration of 1%. Plates were incubated at 37 °C with continuous shaking at 150 rpm for 24 h. MIC value was defined as the lowest phenolic acid concentration at which no visible bacterial growth was observed.

Minimum bactericidal concentration (MBC) was determined by subcultivating 5 µL of each well on brain heart infusion agar (BHI, Biolife, Milano, Italy) in quadruplicate immediately after the MIC assay. Plates were incubated for an additional 24 h at 37 °C, after which bacterial colonies were counted, and the percentage of dead cells was calculated according to the following formula:

$$\mathrm{Dead}\;\mathrm{bacterial}\;\mathrm{cells}\;[\%]\;=\;(1\;-\;\frac{\mathrm{CFU}2}{\mathrm{CFU}1})\times 100,$$

where CFU1 represents the number of bacteria initially added to the wells, and CFU2 represents the number of bacteria that survived phenolic acid treatment.

#### 4.2.4. Influence of Sub-Inhibitory Concentrations of Phenolic Acids on Bacterial Cell Motility

The effect of sub-inhibitory concentrations (SIC) of phenolic acids on the motility of selected soil-derived strains was assessed using a hanging drop assay. Bacterial motility was evaluated before and after the treatment with individual phenolic acids or their combination.

In brief, bacterial strains were cultivated overnight at 30 °C in liquid MHB or YMB, depending on the strain, supplemented with individual or a combination of phenolic acids at SIC (Table A1). Controls included inoculated liquid media without phenolic acids. Following the incubation, hanging drops were prepared and examined under the light microscope (Olympus CX21, Tokyo, Japan) at 1000× magnification with immersion oil. The motility assay following the treatment with sub-inhibitory concentrations of phenolic acids included only naturally motile strains.

### 4.3. Statistical Analysis

All statistical analyses, unless stated otherwise, were performed in R (v4.5.1 [67]). The *A. artemisiifolia* experiment was conducted twice with four replicates each, and analyses were performed on the combined data. Normality and homogeneity of variances were assessed using Shapiro–Wilk and Levene’s tests. Effects of phenolic acids (individual, combinations, and mixtures) and concentrations on radicle length, hypocotyl length, and germination percentage were evaluated using two-way ANOVA, with experimental run as a blocking factor, followed by Tukey’s post hoc test. Dose–response relationships were modeled using log-logistic curves (drc package), and EC_50_ values were estimated. Data for the highest concentrations (15 and 20 mM) of multi-acid mixtures were excluded due to complete inhibition (100%). Germination dynamics were analyzed using three-parameter log-logistic functions (LL.3), estimating t_10_, t_50_, and t_90_ with the ED function [68], and curve differences were tested with the compParm function [68,69]. Since MIC and MBC values are discrete measurements derived from two independent replicates, identical values were observed for each phenolic acid-strain combination. Therefore, MIC and MBC values against soil-derived bacterial strains were reported as single representative values. Differences in antibacterial activity among phenolic acids and their combinations were assessed by rank-based Kruskal–Wallis tests [70] followed by Dunn’s multiple comparisons with Benjamini–Hochberg correction (*p* < 0.05). The figures were produced with the version 4.0.1. [71].

## 5. Conclusions

Our study found that the phenolic acid concentrations needed to inhibit soil-derived strains, except for PPK and MIX6, are generally higher than those required to suppress *A. artemisiifolia* Overall, due to their relatively weaker antibacterial activity, vanillic, *p*-coumaric, and ferulic acids, either alone or in combination, and *p*-hydroxybenzoic acid alone have emerged as the most promising candidates for developing alternative weed control strategies that minimize adverse effects on beneficial soil bacteria, as they are unlikely to harm these organisms when applied under realistic field conditions. The long-term effects on weed survival and reproduction could not be assessed in this in vitro study and should be addressed in future research. Moreover, if phenolic acids are further explored in weed management strategies in combination with reduced herbicide doses, this study clearly demonstrates that chlorogenic and protocatechuic acids can also be used at concentrations below 2.5 mM.

## Figures and Tables

**Table 1:** Minimum inhibitory and minimum bactericidal concentrations of phenolic acids and their combinations against selected soil-derived bacterial strains.

Strain	Identification	MIC (MBC) * [mM]								
		PHBA	PKA	CGA	FA	VA	PCA	PPK	FVP	MIX6
PS01	*Pseudomonas glycinae*	40 ^b^	20 ^ab^	20 ^ab^	40 ^b^	20 ^ab^	40 ^b^	2.5 ^a^	5 ^ab^	2.5 ^a^
PS02	*Pseudomonas chlororaphis*	20 ^bc^	10 ^ab^	20 ^bc^	20 ^bc^	20 ^bc^	40 ^c^	5 ^ab^	10 ^ab^	2.5 ^a^
PS03	*Pseudomonas jessenii*	10 ^abc^	10 ^abc^	10 ^abc^	40 ^c^	20 ^bc^	40 ^c^	5 ^ab^	20 ^bc^	2.5 ^a^
PS04	*Pseudomonas* sp.	10 ^ab^	10 ^ab^	10 ^ab^	40 ^b^	20 ^b^	40 ^b^	2.5 ^a^	10 ^ab^	2.5 ^a^
PS05	*Pseudomonas chlororaphis*	10 ^abc^	10 ^abc^	10 ^abc^	40 ^c^	20 ^bc^	20 ^bc^	5 ^ab^	20 ^bc^	2.5 ^a^
PS06	*Pseudomonas* sp.	10 ^ab^	10 ^ab^	10 ^ab^	40 ^b^	20 ^b^	20 ^b^	2.5 ^a^	2.5 ^a^	2.5 ^a^
PS07	*Pseudomonas chlororaphis*	10 (20) ^ab^	10 (20) ^ab^	10 (20) ^ab^	40 ^b^	20 (40) ^b^	40 ^b^	5 ^a^	10 ^ab^	2.5 ^a^
BC01	*Bacillus licheniformis*	10 ^ab^	10 ^ab^	10 ^ab^	20 ^b^	20 ^b^	20 ^b^	2.5 ^a^	10 ^ab^	2.5 ^a^
BC02	*Bacillus* sp.	10 ^ab^	10 ^ab^	10 ^ab^	20 ^b^	20 ^b^	20 ^b^	2.5 ^a^	10 ^ab^	2.5 ^a^
BC03	*Bacillus pumilus*	20 ^ab^	40 ^b^	10 ^ab^	40 ^b^	20 ^ab^	40 ^b^	5 ^a^	10 ^ab^	2.5 ^a^
BC04	*Bacillus* sp.	10 ^ab^	5 ^ab^	5 ^ab^	40 ^b^	20 ^b^	40 ^b^	2.5 ^a^	10 ^ab^	2.5 ^a^
PB01	*Peribacillus* sp.	20 ^ab^	5 ^a^	20 ^ab^	40 ^b^	20 ^ab^	40 ^b^	5 ^a^	10 ^ab^	5 ^a^
PB02	*Peribacillus* sp.	5 ^ab^	5 ^ab^	5 ^ab^	10 ^ab^	20 ^b^	20 ^b^	2.5 ^a^	10 ^ab^	2.5 ^a^
PB03	*Peribacillus* sp.	40 ^b^	10 ^ab^	10 ^ab^	20 ^ab^	20 ^ab^	40 ^b^	2.5 ^a^	10 ^ab^	2.5 ^a^
PB04	*Peribacillus* sp.	20 ^ac^	10 ^ab^	20 ^ac^	20 ^ac^	20 ^ac^	40 ^c^	2.5 ^a^	10 ^ab^	2.5 ^a^
PB05	*Peribacillus* sp.	20 ^ab^	5 ^a^	10 ^ab^	40 ^b^	40 ^b^	40 ^b^	5 ^a^	10 ^ab^	5 ^a^
PB06	*Peribacillus simplex*	20 ^ab^	2.5 ^a^	2.5 ^a^	40 ^b^	20 ^ab^	20 ^ab^	2.5 ^a^	10 ^ab^	2.5 ^a^
ST01	*Stenotrophomonas humi*	10 ^abc^	5 ^ab^	5 ^ab^	40 ^c^	20 ^bc^	20 ^bc^	2.5 ^a^	10 ^abc^	2.5 ^a^
EB01	*Enterobacter ludwigii*	20 ^ab^	20 ^ab^	40 ^b^	20 ^ab^	40 ^b^	40 ^b^	10 ^a^	20 ^ab^	5 ^a^
EB02	*Enterobacter ludwigii*	40 ^b^	40 ^b^	40 ^b^	20 ^ab^	40 ^b^	40 ^b^	5 ^a^	20 ^ab^	5 ^a^
LE01	*Lelliottia amnigena*	20 ^ab^	20 ^ab^	40 ^b^	20 ^ab^	40 ^b^	40 ^b^	5 ^a^	20 ^ab^	5 ^a^
LE02	*Lelliottia amnigena*	20 ^ab^	10 ^ab^	40 ^b^	10 ^ab^	40 ^b^	40 ^b^	5 ^a^	20 ^ab^	5 ^a^
BR01	*Bradyrhizobium japonicum*	20 ^b^	10 ^ab^	20 ^b^	20 ^b^	20 ^b^	20 ^b^	5 ^a^	10 ^ab^	2.5 ^a^

* MIC—minimum inhibitory concentration; MBC—minimum bactericidal concentration; MIC and MBC values are shown as a single representative value from two identical replicates; MBC values differing from MIC values are shown in parentheses; PHBA—*p*-hydroxybenzoic acid; PKA—protocatechuic acid; CGA—chlorogenic acid; FA—ferulic acid; VA—vanillic acid; PCA—*p*-coumaric acid; PPK—*p*-hydroxybenzoic acid + protocatechuic acid + chlorogenic acid; FVP—ferulic acid + vanillic acid + *p*-coumaric acid; MIX6—*p*-hydroxybenzoic acid + protocatechuic acid + chlorogenic acid + ferulic acid + vanillic acid + *p*-coumaric acid; a–c Different letters within the same row indicate significant differences in the antibacterial activity of a single phenolic acid or phenolic acid combination, based on rank-based Kruskal–Wallis test (*p* < 0.05) followed by Dunn’s post hoc test (*p* < 0.05) against the selected soil-derived bacterial strains.

**Table 2:** Effects of sub-inhibitory concentrations of individual phenolic acids and their combinations on the motility behaviour of selected motile soil-derived bacterial strains; non-motile strains were excluded.

Strain	Identification	Control	PHBA	PKA	CGA	FA	VA	PCA	PPK	FVP	MIX6
PS02	*P. chlororaphis*	+	+	+	+	+	+	+	-	-	-
PS03	*P. jessenii*	+	+	+	+	+	+	+	-	+	-
PS05	*P. chlororaphis*	+	+	+	+	+	+	+	-	+	-
PS06	*Pseudomonas* sp.	+	+	+	+	+	+	+	-	-	-
PS07	*P. chlororaphis*	+	+	+	+	+	+	+	-	+	-
PB01	*Peribacillus* sp.	+	+	-	+	+	+	+	-	-	-
EB01	*E. ludwigii*	+	+	+	+	+	+	+	-	+	-
EB02	*E. ludwigii*	+	+	+	+	+	+	+	-	+	-
LE01	*L. amnigena*	+	+	+	+	+	+	+	-	+	-
LE02	*L. amnigena*	+	+	+	+	+	+	+	-	+	-
BR01	*B. japonicum*	+	+	-	+	+	+	+	-	-	-

Control—inoculated liquid media without phenolic acids; PHBA—*p*-hydroxybenzoic acid; PKA—protocatechuic acid; CGA—chlorogenic acid; FA—ferulic acid; VA—vanillic acid; PCA—*p*-coumaric acid; PPK—*p*-hydroxybenzoic acid + protocatechuic acid + chlorogenic acid; FVP—ferulic acid + vanillic acid + *p*-coumaric acid; MIX6—*p*-hydroxybenzoic acid + protocatechuic acid + chlorogenic acid + ferulic acid + vanillic acid + *p*-coumaric acid. + motile; - non motile.

## Data Availability

Data will be held in a public repository, including URLs, accession numbers or DOIs after acceptance of the paper at: https://dabar.srce.hr/en/browse.

## Appendix A

### Appendix A.1

**Table A1:** Sub-inhibitory concentrations of phenolic acids and their combinations used to assess motility in selected soil-derived bacterial strains.

Strain	Identification	SIC [mM]								
		PHBA	PKA	CGA	FA	VA	PCA	PPK	FVP	MIX6
PS01	*Pseudomonas glycinae*	20	10	10	20	10	20	1.25	2.5	1.25
PS02	*Pseudomonas chlororaphis*	10	5	10	10	10	20	2.5	5	1.25
PS03	*Pseudomonas jessenii*	5	5	5	20	10	20	2.5	10	1.25
PS04	*Pseudomonas* sp.	5	5	5	20	10	20	1.25	5	1.25
PS05	*Pseudomonas chlororaphis*	5	5	5	20	10	10	2.5	10	1.25
PS06	*Pseudomonas* sp.	5	5	5	20	10	10	1.25	1.25	1.25
PS07	*Pseudomonas chlororaphis*	5	5	5	20	10	20	2.5	5	1.25
BC01	*Bacillus licheniformis*	5	5	5	10	10	10	1.25	5	1.25
BC02	*Bacillus* sp.	5	5	5	10	10	10	1.25	5	1.25
BC03	*Bacillus pumilus*	10	20	5	20	10	20	2.5	5	1.25
BC04	*Bacillus* sp.	5	2.5	2.5	20	10	20	1.25	5	1.25
PB01	*Peribacillus* sp.	10	2.5	10	20	10	20	2.5	5	2.5
PB02	*Peribacillus* sp.	2.5	2.5	2.5	5	10	10	1.25	5	1.25
PB03	*Peribacillus* sp.	20	5	5	10	10	20	1.25	5	1.25
PB04	*Peribacillus* sp.	10	5	10	10	10	20	1.25	5	1.25
PB05	*Peribacillus* sp.	10	2.5	5	20	20	20	2.5	5	2.5
PB06	*Peribacillus simplex*	10	1.25	1.25	20	10	10	1.25	5	1.25
ST01	*Stenotrophomonas humi*	5	2.5	2.5	20	10	10	1.25	5	1.25
EB01	*Enterobacter ludwigii*	10	10	20	10	20	20	5	10	2.5
EB02	*Enterobacter ludwigii*	20	20	20	10	20	20	2.5	10	2.5
LE01	*Lelliottia amnigena*	10	10	20	10	20	20	2.5	10	2.5
LE02	*Lelliottia amnigena*	10	5	20	5	20	20	2.5	10	2.5
BR01	*Bradyrhizobium japonicum*	10	5	10	10	10	10	2.5	5	1.25

SIC—sub-inhibitory concentration corresponding to ½ of the minimum inhibitory concentration (MIC); PHBA—*p*-hydroxybenzoic acid; PKA—protocatechuic acid; CGA—chlorogenic acid; FA—ferulic acid; VA—vanillic acid; PCA—*p*-coumaric acid; PPK—*p*-hydroxybenzoic acid + protocatechuic acid + chlorogenic acid; FVP—ferulic acid + vanillic acid + *p*-coumaric acid; MIX6—*p*-hydroxybenzoic acid + protocatechuic acid + chlorogenic acid + ferulic acid + vanillic acid + *p*-coumaric acid.

**Table A2:** Two-way analysis of variance for the reduction (%) of radicle length in *Ambrosia artemisiifolia* L. treated with phenolic acids at different concentrations.

Source of Variability	Df	Sum Sq	Mean Sq	F Value	Pr (>F)
Phenolic acids (PA)	5	59,565.50	11,913.10	61.62	3.35 × 10^−32^
Concentration (C)	6	149,852.58	24,975.58	129.18	2.31 × 10^−51^
PA × C	30	30,982.89	1032.76	5.34	1.06 × 10^−11^
Residuals	126	24,360.89	193.34		

**Table A3:** Effect of treatments on the slope parameter with standard errors, confidence intervals, and CLD grouping. Different lowercase letters represent significant differences (*p* < 0.05) between the phenolic acids.

Treatment	Slope	SE	Lower 95	Upper 95	CLD
CONTROL	−6.7224917	0.50805505	−7.71827962	5.72670384	b
PHBA 10 mM	−2.97460566	0.24042734	−3.44584324	2.50336808	a
MIX6 2.5 mM	−3.05806783	0.22400999	−3.4971274	2.61900825	a
CGA 10 mM	−3.06635038	0.26475618	−3.58527249	2.54742826	a
PKA 20 mM	−3.23327684	0.31097244	−3.84278281	2.62377086	a
MIX6 5 mM	−3.27210686	0.32626096	−3.91157833	2.63263538	a

**Table A4:** Effect of treatments on the maximal germination rate (GRmax) with standard errors, confidence intervals, and CLD grouping. Different lowercase letters represent significant differences (*p* < 0.05) between the phenolic acids.

Treatment	GRmax	SE	Lower 95	Upper 95	CLD
CONTROL	100.154895	0.4297288	99.3126268	100.997164	a
MIX 2.5 mM	90.3804775	0.81948419	88.7742885	91.9866665	b
CGA 10 mM	85.0632916	0.79290011	83.5092074	86.6173758	c
PHBA 10 mM	83.2104613	0.8768939	81.4917493	84.9291734	c
PKA 20 mM	74.1751713	0.76752452	72.6708232	75.6795194	d
MIX6 5 mM	56.365813	1.28405885	53.8490577	58.8825683	e

**Table A5:** Effect of treatments on the area under the curve (AUC) with standard errors, confidence intervals, and CLD grouping. Different lowercase letters represent significant differences (*p* < 0.05) between the phenolic acids.

Treatment	AUC	SE	CLD
CONTROL	4756.47218	17.169759	a
MIX6 2.5 mM	3995.25275	97.1590284	ab
CGA 10 mM	3842.5127	120.758988	ab
PHBA 10 mM	3619.25325	225.885807	a
PKA 20 mM	3347.17622	460.104874	a
MIX6 5 mM	2016.504	143.588379	c
